# Ethnomedical Knowledge of Plants Used for the Treatment of Tuberculosis in Johor, Malaysia

**DOI:** 10.1155/2016/2850845

**Published:** 2016-01-03

**Authors:** Siti Fatimah Sabran, Maryati Mohamed, Mohd Fadzelly Abu Bakar

**Affiliations:** Centre of Research for Sustainable Uses of Natural Resources (CoR-SUNR), Faculty of Science, Technology & Human Development, Universiti Tun Hussein Onn Malaysia (UTHM), 86400 Parit Raja, Batu Pahat, Johor, Malaysia

## Abstract

This study documented ethnomedical knowledge of plants used for the treatment of tuberculosis (TB) and its related symptoms as practiced by the Jakun community of Kampung Peta, situated in Endau Rompin Johor National Park, Johor, Malaysia. Eight key informants were selected by snowball sampling technique and data about medicinal plants were collected by semistructured interviews, participatory observations, and focus group. Qualitative analysis was undertaken using thematic analysis. There were 23 species of plants (22 genera, 20 families) documented and herbarium specimens were deposited at the UTHM Herbarium.* Dipterocarpus sublamellatus* was recorded for the first time with ethnomedical uses while other species were previously reported. The qualitative approach employed in this study demonstrates the emic perspective in terms of perceptions on traditional herbal medicine, transfer of knowledge, significant taboos related with medicinal plants, and their conservation efforts. Local and biomedical terminology in treatment of TB showed substantial correspondence. The outcomes obtained in the study are worth being further investigated for conservation strategies and are worthy of verifying their ethnomedical claims scientifically.

## 1. Introduction

Tuberculosis (TB) is a key global health problem [1]. This ancient, airborne infectious disease is caused by* Mycobacterium tuberculosis* bacterium. In 2012, it is estimated that 8.6 million people developed TB and 1.3 million died from it [1]. Its epidemiology shows that this disease can affect a whole community by causing significant mortality and morbidity to human being [2]. Additionally, occurrence of drug-resistant strains of TB poses serious threat to the current situation and the typical anti-TB drugs have caused ruthless side and adverse effects to the patients [3]. An ideal anti-TB regimen is not yet available to combat the resistant strains of TB and the recommended treatment regimens are problematic [4]. Since Johor, having the second highest prevalence of tuberculosis (TB) cases in Peninsular Malaysia, is also home to the Jakun, perhaps documenting the existing ethnomedical knowledge of the Jakun of the treatment for TB and its symptoms could be a leading way towards future discovery of medication for TB [5]. Therefore, the search for at least one potentially new drug derived from nature should be initiated [6]. In this case, ethnomedical knowledge of the Jakun community could provide a lead in primary screening of potential anti-TB agents.

**Figure 1:**
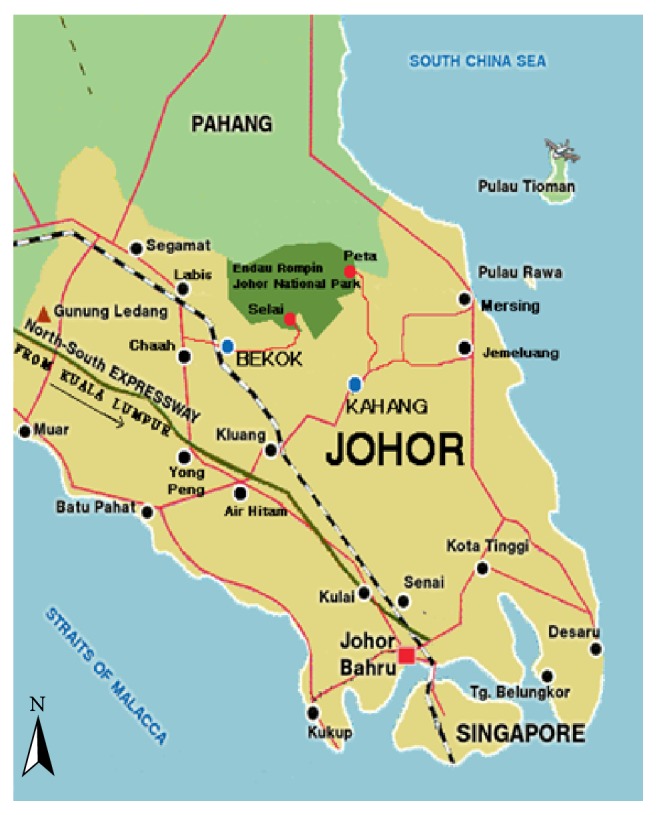
Location of Endau Rompin Johor National Park and Kampung Peta [11].

Malaysia is ranked as the twelfth megadiverse country in the world due to its richness and endemism of flora and fauna [7]. Peninsular Malaysia has been estimated to have more than 2,000 species of medicinal plants and there are about 200 species being used by different ethnic groups all around the country [8]. Endau Rompin forest (2°25′12.94′′N, 103°15′40.94′′E) is one of the few remaining areas of virgin lowland rainforest in the southern part of Peninsular Malaysia. Geographically, it is the mainland Asian's southernmost stretch of tropical rainforest. In 1993, 48,905 hectares of the Endau Rompin forest was gazetted as a national park by the state government of Johor [9]. Kampung Peta is a village located outside the boundaries of the park (Figure 1). It has become the main entrance to Endau Rompin Johor National Park in the municipality of Mersing, Johor. Within the rich lowland mixed dipterocarp forest of the park lie various species of plants that provide substantial sources for food, medicines, shelters, timber products, and many more to the nearby civilization [10].

*Orang Asli* is a local term for indigenous people in Peninsular Malaysia. A total of 18 tribes of* Orang Asli* are estimated to be 150,000 people, covering 0.5% of the whole Malaysian population [12]. They are clustered into three major groups: Negrito (northern region), Senoi (middle region), and Proto-Malay (southern region). A tribe called the* Jakun* is a subgroup of Proto-Malay and is the most dominant* Orang Asli* tribe in Johor [13]. The Jakun community of Kampung Peta are descendants of the first inhabitants of Endau River valley [14]. The population of Jakun community in Kampung Peta is about 220 people with 67 households, which represents 2% of the whole* Orang Asli* in Johor [15]. They are still practicing traditional lifestyle amidst modern facilities and strongly adhere to their ancestors beliefs as their way of life. They speak the Jakun dialect which is a subdialect of the Malay language. Their livelihood relies heavily on natural resources around them, which includes combination of fishing, hunting, farming, and trading forest products. Recent years have shown tremendous changes in their lifestyle. Due to socioeconomic improvement, the younger generation of Jakun are able to get higher education and many have migrated to other places [16].

The first documentation work about medicinal plants used by the Jakun community in Kampung Peta recorded 52 plants species used for minor common ailments [17]. Additionally, 118 species of plants from Endau Rompin Johor National Park were also surveyed against their alkaloid, saponin, triterpene, and steroid contents [18]. Recent inventory data taken from 2005 to 2008 showed that approximately 54 nontimber plant families are used for various ethnobotanical uses that include Rubiaceae (16 species), Arecaceae (12 species), Annonaceae (9 species), Melastomataceae (7 species), Euphorbiaceae (5 species), Leguminosae and Zingiberaceae (4 species), and Connaraceae, Liliaceae, Myrtaceae, Rhizophoraceae, and Piperaceae (3 species) [19–21]. This information demonstrates the prominence and dependency of the Jakun community on such plants that may have potential value as sources of active medicinal principles. Although the Jakun community in Kampung Peta still depend on their ethnomedical knowledge for primary healthcare, it is easy to lose this attribute as the world progresses towards modernization. Therefore, by conducting proper documentation, existing ethnomedical knowledge could be maintained and not easily manipulated.

This paper aims to document plants used for the treatment of TB and its related symptoms guided by ethnomedical knowledge of the Jakun community in Kampung Peta, Johor, south of Peninsular Malaysia. To the best of our knowledge, no previous ethnomedical study had been conducted specifically for treatment of TB from this community. The new information gained from this study might initiate further studies to aim at exploring the anti-TB potentials of the plants, supporting the sustainability of traditional herbal medicine in local community, and conserving plants diversity.

## 2. Methodology

### 2.1. Ethical Authorizations

Following ethical guidelines from [22], approval from Department of Orang Asli Development (JAKOA) under the Malaysia Ministry of Rural and Regional Development was acquired. Plants were collected under a permit approved by Johor National Parks Corporation (JNPC). Written Prior Informed Consent (PIC) was obtained and Access and Benefit Sharing (ABS) was explained during data collection. The inclusion of the headman (*tok batin*) of the Jakun of Kampung Peta indicated our commitment in fulfilling ABS mechanism at this juncture of time. Participation of informants was dependent on their self-willingness and acceptance of the terms in PIC and ABS, which were specially developed for this research program.

### 2.2. Data Collection

Fieldworks were conducted between April 2013 and April 2014. Key informants were recruited using snowball sampling method [23–25]. Initially, a courtesy call was made to the village headman. The purpose of the research was briefed to him and he then assigned his people to participate in the interviews. During the discussion with the first informant (R1), she then referred to other informants. Criteria of selection were also based on (i) the recognition that they are local practitioners by the Jakun community, (ii) their ability to identify plants and explain the uses, and (iii) the recommendation by park officers for their involvement in traditional herbal medicine. Eight key informants of Jakun community were selected from Kampung Peta as shown in Table 1. Each informant has vast experience in the areas of traditional practices, herbal formulations, field identification, and collection of medicinal plants. One of the informants (R8) was selected for her experience in preparing herbal remedies to treat her son who claimed to have active TB and now recovered from it.

In-depth, semistructured interviews were carried out as guided [26, 27]. The interviews were comprised of three parts: (i) demographic profile of the informants such as name, gender, age, marital status, religious belief, how they gained the knowledge, duration of practice, education level, and occupation; (ii) information about medicinal plants consumed by the Jakun related to signs and symptoms of TB (cough, cough with blood, cough with sputum, fever, night fever, loss of weight or appetite, asthma, rheumatism, and fatigue), including the local names, parts used, method of preparation, dosage, and administration; and (iii) significant aspects of Jakun's ethnomedical knowledge such as beliefs or taboos related to the plants. Each interview session lasted an average of two hours, ranging from 30 minutes to three hours.

Participatory observations were also done during casual or social meetings for any occurrences of what related to ethnomedical knowledge of plants among the Jakun community. This also created a unique opportunity for the main researcher to get closer, to build up rapport and trust, and to minimize the cultural gap between the main researcher and the informants.

A 2-day training course on “*Documentation of Ethnobotanical Knowledge of Indigenous People*” was organized by Universiti Tun Hussein Onn Malaysia (UTHM) to form a focus group. The objectives for this course were to provide training on ethnobotanical documentation and to establish an open-ended discussion among researchers, state agencies, and four representatives from the Jakun community. The discussion session was directed to encourage the representatives to share and discuss their knowledge in greater depth. Questions like “How do you feel about your mom using herbs? Do you think it is ancient or out-dated?” were asked. In this way, the representatives were able to provide in-depth answers as individuals.

### 2.3. Plants Identification

Plant samples were collected following the standard guidelines with consideration to the conservation of the species [28]. Triplicates of each Herbarium specimens were pressed, oven-dried at 40°C for two weeks, and mounted on Herbarium sheets, which were then deposited at Universiti Tun Hussein Onn Malaysia (UTHM) Herbarium Collection for future references. Other standard data such as location, vegetation, habitat description, other medicinal plants present, and local plant name were recorded at each field site on preprepared forms. Digital photographs showing morphological features were also taken. The prepared specimens were compared to previously identified specimens from Kepong National Herbarium (KEP). The authentication was done by Kamarudin Saleh from Forest Research Institute Malaysia (FRIM).

### 2.4. Data Analysis

Tables and graphs were generated in standard software, namely, Microsoft Excel 2013 [29]. Data from the transcribed interviews were analysed qualitatively following the emic approach [30]. Thematic analysis, which was derived from informants' own concepts, was applied to conceptualize the data, identify themes, and assign concept codes [31]. Reported uses of various medicinal plants were compared with previously published ethnomedical literatures about medicinal plants in Endau Rompin Johor National Park to cross-check and identify new medicinal uses [32] and any loss of knowledge [33].

## 3. Results and Discussion

### 3.1. Demographics

The eight key informants were two males and six females, with ages ranging between 40 and 66 years. In common, they were individuals who gained knowledge of medicinal uses of plants from self-experiences and observations and through their parents as detailed in Table 1. Additionally, some of them were formally trained by a local herbalist due to their occupational requirement as park staffs. The God, forest spirits, or deceased ancestors revealed the knowledge through dreams, as experienced by one of the informants' son. This showed that belief and ethnomedical knowledge were integrated in this study. Although they were not regarded as the local experts or herbalists, they were the traditional herbal medicine practitioners that would genuinely describe the plants they were very familiar with to the researcher. Additionally, the main advantage of employing the snowball sampling method was that the subsequent key informants were introduced to the researcher based on acknowledgement by their own tribe. Thus, in this study, characteristics such as age, gender, marital status, belief, and education level did not influence the acquisition of their ethnomedical knowledge of plants.

### 3.2. Ethnomedical Knowledge of Plants

#### 3.2.1. Plant Families, Habitat

The ethnomedical knowledge about the plants was summarized in Table 2. A total of 23 species of medicinal plants were documented in this study. From Table 2, 22 genera and 20 botanical families were presented, indicating that the medicinal plants were much diversified taxonomically. The top most represented families were Arecaceae, Aristolochiaceae, and Rubiaceae with two species each of the total distribution. Others were the remaining 17 families (Loganiaceae, Musaceae, Cucurbitaceae, Sterculiaceae, Annonaceae, Dipterocarpaceae, Dilleniaceae, Hypoxidaceae, Myrtaceae, Nepenthaceae, Urticaceae, Simaroubaceae, Euphorbiaceae, Poaceae, Anacardiaceae, Ebenaceae, and Connaraceae), which represented only one species each.

The plant families consist of various habitats such as trees (7 species), climbers (7 species), shrubs (4 species), herbs (3 species), and hemiepiphyte (1 species). In this study, the significant uses of the climbers in the Jakun ethnomedical knowledge showed a substantial relationship between traditional knowledge and plant conservation. As examples, the climbers are greatly dependent on large trees to grow and survive and vice versa [34, 35]. At the same time, the climbers play an essential role as remedial resource to the local community. Uncontrolled logging and deforestation could cause threats to the species of climbers and eventually erode local knowledge about medicinal plants [36]. Therefore, not only is documenting ethnomedical knowledge of plants an inventory* per se*, but it also contributes to the issue of biodiversity conservation threats such as deforestation, habitat modification, and unsustainable overexploitation.

#### 3.2.2. Symptoms of TB

The 23 medicinal plants species recorded in this study were used to treat an active TB disease (claimed by the Jakun community) and nine of TB-related symptoms. The most frequently cited medicinal plants were used for fever (30%) as it is a common ailment even in other communities. Following that is cough (22%), fatigue (17%), and asthma (13%). 9% of the species were used to treat cough with blood, night fever, cough with sputum, and rheumatism, whereas 4% were documented to treat active TB and loss of appetite.

#### 3.2.3. Parts Used

In this study, various plant parts were used for the herbal preparation. Commonly, roots and stems were used and this applied to 39% of all plants listed. This is followed by shoots involving 9%. The least used parts were flowers, fruits, seeds, and stem barks, for 4% of listed plants. According to informants, the root is the main plant part used in the Jakun traditional medicine. This may arise from the fact that the roots act as reservoirs for water and mineral uptakes, which is rich with variety of secondary metabolites such as steroids, alkaloids, terpenes, and volatile organic compounds [37]. 83% of the documented species were used individually, while the remaining 17% were recommended to be used in mixtures.

#### 3.2.4. Preparation and Administration

In the Jakun community, herbal remedies are usually prepared fresh. If this is not the case, they will dry the plant parts (usually the roots) and keep them in a proper storage before use. The most common method of preparation was decoction in water (43% of listed plants) followed by collection of sap (35%) and being eaten raw (13%). The less common methods include infusion in water and being cooked as food (9% each) and maceration in water and decoction in oil (4% each). Decoction in water is equivalent to aqueous extraction and it appears to be much favoured because it is easier to prepare. Additionally, water is the best solvent to dissolve hydrophilic compounds that are responsible for various antimicrobial activities [38]. In this study, the most typical way of administration was taken in a form of drink (83%) followed by taken as food (17%) and applied on tongue (9%). The least typical ways of administration were as massage oil and cold press and for bathing (4% each). These elements might explain the relatively good association between preparation and administration of herbal remedies, and more than three-quarters of the listed plant species (87%) were taken orally as compared to those taken for topical applications (4%).

#### 3.2.5. Conservation Status

22 species of the plants documented in Table 2 are taken from the wild, whereas only one species (*Gardenia* sp.) is cultivated. Medicinal plants are generally harvested from nearby forest areas by the local people. These results corroborate the ideas of Ceuterick et al. [31], who suggested that local people use herbal remedies that are readily available and easily accessible in the natural vegetation around their settlement. However, forestry overexploitation for timber products [39, 40] and wide popularity of their local use lead to overharvesting [41] and perhaps put them into higher risk of extinction in the future if no conservation efforts are engaged. In this study, all informants showed an understanding about conservation practices. Their strong affection towards the forest was observed by the researcher during the fieldworks. The implementation of* ex situ* conservation through home garden and* in situ* conservation through the establishment of ethnobotanical garden in national park area was efforts made by the Jakun community and the national park authority.

If I get medicines that are rare… highly healing… I will plant them. (R1, 2014, personal communication)

It is interesting to note that Jakun's ethnomedical knowledge reflected their thoughtful conservation efforts and respects towards nature. Apart from replanting the medicinal plants, they also practice to reuse the raw materials.

I will not waste the materials. After using, I collect the decoction and I dry the remaining materials again to reuse them. (R6, 2014, personal communication)

Perhaps, unintentionally, these ethnomedical practices that implement sustainable method of harvesting have contributed to the conservation of medicinal plants. In addition, the awareness of loss of herbs among the Jakun community shows that the natural resources are increasingly threatened and intensifying efforts need to be implemented immediately to curb this problem. One of the informants stated that majority of the medicinal plants are easily available but certain species are also available with difficulty.

Before this it was very easy to find. Now, it is hard. (R4, 2014, personal communication)

Pardo-de-Santayana and Macía [42] agreed that local resources particularly the plants they use as food and medicine are crucial to ensure that those communities can continue to live and benefit from their local ecosystems in a sustainable way.

#### 3.2.6. Frequency of Citation

The plants with the highest frequency of citation by informants are* Strychnos ignatii* and* Calamus* sp. (6 citations), whereas plants with the lowest frequency of citation by the informants are* Diospyros cauliflora* and* Rourea mimosoides* (1 citation). Even though six species were cited by less than three informants (*n* < 3), their medicinal uses appear to be worthy of further investigations to verify their possible pharmacological activities especially those used to treat constitutional symptoms of TB such as night fever and cough with sputum [30]. However, being named by at least three informants (*n* ≥ 3) is the most typical cut-off point used by ethnobotanists to establish agreement [43].

#### 3.2.7. Novel Knowledge

Comparison with previous documentation works appeared to suggest that this study attained one new ethnomedical knowledge and one new claim. Majority of the species reported by the informants were already known as medicinal plants in Malaysia except for* Dipterocarpus sublamellatus*. Therefore, in this study,* D. sublamellatus* was documented for the first time with ethnomedical knowledge while the rest of the listed species were formerly reported with diverse medicinal uses from other indigenous communities.* D. sublamellatus* was specifically used to treat active TB as claimed by some of the key informants. It is interesting to note that this particular species is a member of Dipterocarpaceae family, which was reported to contain sesquiterpenes, triterpenes, coumarin derivatives, phenolics, essential oil, and isoquinoline alkaloids groups [44, 45]. The use of* Gardenia* sp. as medicinal plants for the Jakun community, which was another new claim recorded in this study, was not previously recorded. The knowledge might be gained by cross-cultural interaction with outsiders like the Malays and Chinese, as it is typical ornamental and medicinal plant in these cultures [46].

### 3.3. Thematic Analysis

The thematic analysis approach was helpful to recognize the culturally valuable ethnomedical knowledge of the Jakun community. Repetition of certain words provided a cue to assign coding and identify themes. In addition, the repetition of questions was deliberated to provide a focus for analysis. For instance, the word “time” appeared frequently during the interviews in describing events of plant collection and herbal administration. Subcodings such as “collecting” or “eating” would be a reference to a theme such as “taboos.” Once the themes emerged, data were fragmented to lift coded elements out of the context of each interview to list comments and information by group [24, 25].  Table 3 lists the themes that emerged from the codings.

#### 3.3.1. Perceptions on Traditional Medicine

Medicinal plants were fairly important in the Jakun community for both the elderly and the young generations. The use of traditional medicine did not seem to conflict with the use of modern medicine. In many cases, they complemented each other. However, there were some contraries among the elder and younger generations of Jakun community in Kampung Peta. As examples consider the following:

We never abandon our traditional practices. Just like you, the Malay; if you don't get well surely you will go to the hospital. We still carry out as what our ancestors have been practicing before and never leave it behind. (R1, 2014, personal communication)

“If modern medicine is not effective, I have to look for forest remedies as an alternative.” (Son of R1, 2014, personal communication)

The elder generation uses traditional herbal medicine as the primary source of healthcare while the younger generation uses traditional herbal medicine as the alternative source of healthcare if the modern medicine seems not effective. From the focus group discussion, Jakun's representative expressed his feelings of being the young generation of Jakun who is keeping up with the modern lifestyle and his effort to preserve their traditional knowledge. He mentioned the following:

I do not feel ashamed to the fact that my mother is practicing traditional herbal medicine. Indeed, I feel so proud of it. I also want to learn about it and use it to my daughter. (Rudi bin Kudi, 2013, personal communication)

Although the elder generations are practicing less frequently ethnomedicine due to modernization, such declaration as above proved that the younger generations of the Jakun in Kampung Peta are still supporting the strong practices of ethnomedical knowledge of their ancestors.

#### 3.3.2. Transfer of Knowledge

During present study, it was found that the knowledge about utilization of medicinal plant species is generally accumulated by observation and experiences and transferred orally to the next generation without any systematic process. However, it is certain that such knowledge system is at the risk of fading in the future [47]. Lack of interest from the youth is one of the main concerns among the elderly. The young generation of Jakun shows less attention and are not keen on learning their traditional knowledge from the elder generation. A likely explanation is that because it has little scope for money. Therefore, they engage themselves in other occupations [48]. One of the informants narrated the following:

Even so… the community… mostly the new generation could not recognize the medicinal plants. This is why I tell them, they are the local people but they do not recognize the cures from the forest. (R1, 2014, personal communication)

Commitment towards other responsibilities such as seeking formal education was given more priority compared to learning and teaching about ethnomedical knowledge. The informant explained the following:

How can we teach our grandchildren about this knowledge while they are studying at school? (R4, 2014, personal communication)

Assimilation to modern lifestyle by the young generation most probably contributes to the huge impact on transfer of knowledge. At the time this study was conducted in 2013, the community in Kampung Peta had already gained access to modern medical treatment that was frequently used. It was in the form of a small clinic built by the government in the village to routinely monitor health status of the Jakun community. In addition, they received regular biweekly visits by the medical officers. Moreover, it takes only two hours by car or motorcycle from the village to Mersing Hospital, where doctors are available. Consequently, all of these lessen the exposure to ethnomedical knowledge as a source of remedies [49].

Despite the challenges in transferring the knowledge, having a family and being a parent lead to the awareness in learning about traditional herbal medicine.

He (referring to her son)… now knows a little about forest remedies; after he has a daughter. A few years back before he could not tell anything at all. He definitely knew nothing. Just after his daughter was sick, he asked my opinion on which forest remedies are better. (RI, 2014, personal communication)

Medicinal plants have traditionally been used at home to treat family sickness. In this case, women have particular roles in transferring the ethnomedical knowledge in their capacities as mothers [50].

#### 3.3.3. Taboos Associated with Medicinal Plants

In Jakun's ethnomedical knowledge practices, a few conditions must be followed during the plant collection, preparation, and treatment to ensure efficacy. For medical purposes, medicinal plants should be collected in certain settings such as during the full moon or early in the morning. Indeed, time of harvest is a possible source of variation for the bioactivity of the extracts [51]. They are particularly prohibited to collect plants during “*hujan panas*” or summer rain. They believe that summer rain brings harmful effects on the collector's health and the plants might contain toxic metabolites. Additionally, they are aware of the safety and dosage issues particularly if they take traditional medication together with modern medicine. As demonstrated in Table 2, most of the medicinal plants are prepared as water infusion. The water infusion mainly extracts bioactive compounds such as anthocyanins, tannins, saponins, and terpenoids [52]. As a result, the herbal preparation should only be taken after meal and the Jakun community would avoid any acidic or spicy food during treatment to avoid stomach pain.

#### 3.3.4. Confusion in Names

Confusion of plant names and terminologies and the appearance of uncertainties as shown in Table 2 indicate the erosion of ethnomedical knowledge among the Jakun and this was apparent in this study. Khuankaew et al. [49] suggested that lack of experience with the ethnomedical knowledge practices, which is very vital in the transmission of knowledge, might be a possible factor. This event also suggests that certain knowledge might potentially be lost as a form of deculturation. The reason as to why the Jakun people stop using certain remedies may be due to availability of better alternatives (modern medicine). Ceuterick et al. [31] concluded that herbal remedies can function as ethnic markers. Thus, erosion of this traditional knowledge and practices may possibly weaken Jakun's sense of identity.

During the interviews, some of the informants gave information about the plants that they previously consumed themselves. On the other hand, some of the informants gave information about medicinal plants that they thought the researcher might be interested in although they have little knowledge about the plant. It is important to bear in mind the possible bias in these responses. Hence, confirmation using quantitative approach should be employed to ratify the statement and to eliminate bias of information.

### 3.4. Correspondence between Local and Biomedical Terminology

Following their emic perceptions, all of the key informants were able to differentiate the symptoms of TB as described by the researcher and to define their ethnomedical terms according to their understanding.  Table 4 lists the symptoms of TB given by informants and their equivalent biomedical terms.

Based on Table 4, 14 local terms of TB-related symptoms were listed and each term was capable of being translated into standard biomedical terms. Terminology is one of the challenges during ethnomedical knowledge documentation [51, 52]. Mcclatchey [53] emphasized that it is critical to use terms that are meaningful within a community, even if they are obscure to scientific fields. This is because culture defines medicine while disease etiologies differ between ethnomedical systems [54]. As the one discussed here, Heinrich et al. [55] argued that translating indigenous and local diagnosis into biomedical terms is ideally essential for future clinical assessment.

In the Jakun community, TB is closely associated with black magic. “*Hasad dengki*” or jealousy was speculated as the cause for this disease. According to the informant,

in our community, this disease is typically linked to jealousy. It is intended to destroy the person. We no longer practice such custom and I, myself prohibit it. (Sangka Chuka, 2013, personal communication)

In Kampung Peta, “*bomoh*” could also be consulted to cure less acute conditions by employing his knowledge of the chemical properties of plants. Therefore, any plants might be used as a medicinal plant with some addition of charm or “*jampi*.” Some of the plants introduced by the “*bomoh*” as medicinal plants might over time be incorporated into the group of medicinal plants used by common people in the village. Additionally, the Jakun community also believed that, other than the “*bomoh*,” any selected individual could receive knowledge about forest remedies through dreams revealed by the spirits of the jungle (*semangat hutan* or* dewa*) or their deceased ancestors, who sympathize with their sufferings. Additionally, the Jakun community have not entirely stopped believing in black magic and the powers of plants to impose curses to cure or neutralize curses. But as the Malays and Chinese came in they perceive all black magic as the work of demons and it should be strictly avoided. They still practice animism and believe that God has given plants their specific qualities and their power to act as remedies.

## 4. Conclusions

This study has contributed to the scientific documentation of medicinal plants used for the treatment of TB in Johor, Malaysia. The 23 species of medicinal plants recorded in this study demonstrate that the Jakun community in Kampung Peta are still rich in ethnomedical knowledge particularly of treatment of TB and its related symptoms. The most frequently cited species were* Strychnos ignatii* and* Calamus* sp.* Dipterocarpus sublamellatus* was recorded for the first time for its ethnomedical knowledge and traditionally claimed to treat active TB by the Jakun. While other species were formerly reported,* Gardenia* sp. was a new addition to Jakun's ethnomedical knowledge. Jakun's ethnomedical knowledge needs to be conserved as the larger percentage of the traditional practitioners is older generation and some of the knowledge was apparently eroded in this study. The qualitative approach employed in this study successfully provide the emic perspective in terms of perceptions on traditional herbal medicine, transfer of knowledge, significant taboos related with medicinal plants, and their conservation efforts. Local people and biomedical terminology in treatment of TB showed substantial correspondence. Further studies are in progress on the antituberculosis assay to validate their traditional claims.

## Figures and Tables

**Table 1:** Characteristics of the selected key informants in Kampung Peta.

Code	Gender	Age	Marital status	Belief	Knowledge gained from	Duration of practice	Education level	Occupation
R1	F	66	Widowed	A	S, O, P, and H	Since small	Primary school	Retired park staff, farmer
R2	F	57	Married	A	S, O, P, and H	Since small	No formal education	Retired park staff, farmer
R3	M	58	Married	A	S, O, and P	Since young	No formal education	Handicraft, herbal, and forest products entrepreneur
R4	F	55	Married	A	S, O, P, and H	After being married	No formal education	Park staff, farmer
R5	F	40	Single	A	S, O, P, and H	Since young	No formal education	Park staff
R6	F	44	Married	I	S, O, P, and H	Since small	Primary school	Park staff, trade forest products
R7	M	45	Single	A	S, O, and P	Since small	No formal education	Park staff
R8	F	55	Married	A	S, O, and G	3 months	Primary school	Park staff

Codes R1–R8 refers to informant's name. R1: Dido Lanau, R2: Lindan Jala, R3: Awang Kudi, R4: Kikai Akar, R5: Resnah Jala, R6: Azizah Hussien, R7: Salam Liman, R8: Kechek Chuka, F: female, M: male, A: animism, I: Islam, S: self-experienced, O: observation, P: parents, H: herbalist, and G: God or spirit.

**Table 2:** List of medicinal plants recorded in this study.

Botanical information	Symptoms	Parts used	Methods of preparation	Ways of administration	Frequency of citation	Source of plants
*Strychnos ignatii* Berg. Akar Ipoh Loganiaceae Climber SUNR(P)001	Fever, rheumatism	Stem	Decoction in water, infusion in water	Oral: drink	6	The wild

*Calamus *sp. Rotan sepetang Arecaceae Climber SUNR035	Fever	Stem	Sap collected	Oral: drink	6	The wild

*Calamus scipionum* Lour. Rotan semambu Arecaceae Climber SUNR040	Fever	Stem	Sap collected	Oral: drink	5	The wild

*Musa gracilis* Holttum Pisang sum Musaceae Herb SUNR003	Cough	Stem, flower	Sap collected	Oral: drink, applied on tongue	5	The wild

*Thottea praetermissa *T.L. Yao Perut keletong Aristolochiaceae Shrub SUNR034	Cough, cough with sputum	Root	Decoction in water, raw	Oral: drink, eaten raw	5	The wild

*Hodgsonia macrocarpa *(Blume) Cogn. Teruak Cucurbitaceae Climber SUNR001	Fever	Stem	Sap collected	Oral: drink	4	The wild

*Scaphium macropodum* (Miq.) Beumée ex. Heyne Kembang semangkok Sterculiaceae Tree SUNR021	Fever (high)	Seed	Infusion in water	Oral: drink, mucilage eaten	4	The wild

*Polyalthia bullata* King Tungkat Ali Hitam Annonaceae Shrub SUNR030	Fatigue	Root	Decoction in water	Oral: drink	4	The wild

*Dipterocarpus sublamellatus* Foxw. Keruing air Dipterocarpaceae Tree SUNR037	TB	Stem bark	Decoction in water, decoction in oil	Oral: drink. Topical: massage oil, for bathing	4	The wild

*Tetracera macrophylla* Wall. ex. Hook.f. & Thomson Empelas Dilleniaceae Climber SUNR002	Night fever	Stem	Sap collected	Oral: drink	3	The wild

*Molineria latifolia* (Dryand.) Herb. ex. Kurz var. *latifolia* Lembak Hypoxidaceae Herb SUNR014	Loss of appetite	Fruit	Raw	Oral: eaten raw	3	The wild

*Rhodamnia cinerea* Jack Pelonggot Myrtaceae Tree SUNR019	Fever, fatigue	Stem	Sap collected	Oral: drink	3	The wild

*Nepenthes ampullaria* Jack Sentoyot Nepenthaceae Climber SUNR024	Asthma, rheumatism	Root	Decoction in water	Oral: drink	3	The wild

*Poikilospermum suaveolens *(Blume) Merr. Demom malam Urticaceae Hemi-epiphyte SUNR026	Night fever	Stem	Sap collected	Oral: drink	3	The wild

*Eurycoma longifolia *Jack Tungkat Ali Putih Simaroubaceae Tree SUNR029	Fatigue	Root	Decoction in water. In combination with *Rennellia elliptica, Polyalthia bullata, *and others	Oral: drink	3	The wild

*Gardenia* sp. Bunga cina Rubiaceae Shrub SUNR020	Fever	Shoot, leaf	Maceration in water	Topical: cold press	3	Cultivated

*Macaranga gigantea *(Rchb.f & Zoll.) M.A. Tudung Euphorbiaceae Herb SUNR005	Cough	Stem	Sap collected	Oral: applied on tongue	2	The wild

*Leptaspis urceolata* (Roxb.) R.Br. Lapun puyuh Poaceae Herb SUNR012	Asthma, cough with sputum	Root	Decoction in water	Oral: drink	2	The wild

*Thottea grandiflora* Rottb. Hempeduk beruang; Telingok kelawar Aristolochiaceae Shrub SUNR022	Cough, asthma	Root	Decoction in water	Oral: drink	2	The wild

*Campnosperma auriculatum* (Blume) Hook.f. Habong Anacardiaceae Tree SUNR028	Cough with blood	Shoot, root	Decoction in water, raw, and cooked	Oral: drink, eaten raw, and cooked as food	2	The wild

*Diospyros cauliflora* Blume Uncertain^*∗*^ Ebenaceae Tree SUNR013	Cough	Uncertain^*∗*^	Uncertain^*∗*^	Uncertain^*∗*^	1	The wild

*Rourea mimosoides* (Vahl.) Planch. Pengesep Connaraceae Climber SUNR033	Cough with blood	Root	Decoction in water	Oral: drink	1	The wild

^*∗*^The informants were unable to provide the detailed information regarding the criteria for this plant.

**Table 3:** Themes that emerged via the coding process.

Themes	Subthemes	Codings
Perceptions on traditional medicine	Primary source of healthcare for elder generation	Primary
	Alternative source of healthcare for younger generation	Alternative

Transfer of knowledge	Mothers have a significant influence	Mothers
	The young generation are not interested to learn traditional knowledge due to modern lifestyle	Time

Conservation of medicinal plants	Some valuable and in-demand herbs are difficult to find	Difficult to find
	The location to collect plants is far	Too far
	They use only small amount, use them when necessary, and reuse the materials	Reuse
	They plant the seedlings	Replant

Taboos	Avoid taking prohibited meals during treatment	Eating
	Nice weather is a good time	Collecting

Confusion of names	Appearance of uncertainties	Names

**Table 4:** Symptoms of TB given by informants and their equivalent biomedical terms.

Ailment categories	Biomedical terms	Local terms
Respiratory diseases and fever	Cough	*Se'eh, batuk, gatal-gatal tekak, sakit lidah*
	Cough with sputum	*Batuk berkahak*
	Ordinary fever	*Demam, panas dalam*
	High fever	*Demam panas*
	Asthma	*Semput *
	Chest pain	*Sakit dada*
	Night fever	*Demam malam*

Arthritis	Rheumatism	*Sakit dalam badan*
	Joint pain	*Sakit lutut, sakit sendi*

Ear, nose, throat bleedings	Nose	*Hidung dan tekak berdarah*
	Sore throat	*Sakit leher*
	Cough with blood	*Batuk berdarah*

Others	Fatigue	*Lemah badan*
	Loss of appetite	*Kurang/Tiada selera makan*
